# A Four-Inflow Construction to Ensure Thermal Stability and Uniformity during Hyperthermic Intraperitoneal Chemotherapy (HIPEC) in Rats

**DOI:** 10.3390/cancers12123516

**Published:** 2020-11-26

**Authors:** Daan R. Löke, Roxan F. C. P. A. Helderman, Jan Sijbrands, Hans M. Rodermond, Pieter J. Tanis, Nicolaas A. P. Franken, Arlene L. Oei, H. Petra Kok, Johannes Crezee

**Affiliations:** 1 Department of Radiation Oncology, Amsterdam University Medical Centers, University of Amsterdam, Cancer Center Amsterdam, Meibergdreef, 1105 AZ Amsterdam, The Netherlands; d.r.loke@amsterdamumc.nl (D.R.L.); f.c.helderman@amsterdamumc.nl (R.F.C.P.A.H.); j.sijbrands@amsterdamumc.nl (J.S.); h.rodermond@amsterdamumc.nl (H.M.R.); n.a.franken@amsterdamumc.nl (N.A.P.F.); a.l.oei@amsterdamumc.nl (A.L.O.); h.p.kok@amsterdamumc.nl (H.P.K.); 2 Department for Experimental Oncology and Radiobiology (LEXOR), Center for Experimental and Molecular Medicine (CEMM), Amsterdam University Medical Centers, University of Amsterdam, Cancer Center Amsterdam, Meibergdreef, 1105 AZ Amsterdam, The Netherlands; 3 Department for Surgery, Amsterdam University Medical Centers, University of Amsterdam, Cancer Center Amsterdam, Meibergdreef, 1105 AZ Amsterdam, The Netherlands; p.j.tanis@amsterdamumc.nl

**Keywords:** peritoneal metastasis, cytoreductive surgery, hyperthermic intraperitoneal chemotherapy (HIPEC)

## Abstract

**Simple Summary:**

We developed and validated a preclinical in vivo hyperthermic intraperitoneal chemotherapy (HIPEC) setup using state-of-the-art techniques in rats, including dedicated treatment planning tools and a computer-aided design to achieve well-controlled homogeneous peritoneal flow. Our four-inflow construction resulted in more stable and homogeneous thermal distributions than using a one-inflow construction, with lower standard deviations and less thermal losses. Core temperatures were kept stable using occasional tail cooling, and rarely exceeded 39 °C. This validated design can improve accuracy in future in vivo experiments investigating the impact of relevant treatment parameters on the efficacy of different HIPEC protocols such as: drug type, temperature and duration.

**Abstract:**

Background: Hyperthermic intraperitoneal chemotherapy (HIPEC) after cytoreductive surgery (CRS) is used for treating peritoneal metastases of various origins. Present HIPEC protocols have rarely been validated for relevant parameters such as optimal agent, duration and perfusate temperature. In vitro experiments are not completely representative of clinical circumstances. Therefore, a good preclinical in vivo HIPEC model is needed in which temperature distributions can be well-controlled and are stable throughout treatments. Methods: We designed a setup able to generate and maintain a homogeneous flow during a 90-min HIPEC procedure using our in-house developed treatment planning tools and computer aided design (CAD) techniques. Twelve rats were treated with heated phosphate-buffered saline (PBS) using two catheter setups (one vs. four- inflows) and extensive thermometry. Simulated and measured thermal distribution and core temperatures were evaluated for the different setups. Results: Overall, the four-inflow resulted in more stable and more homogeneous thermal distributions than the one-inflow, with lower standard deviations (0.79 °C vs. 1.41 °C at the outflow, respectively) and less thermal losses. The average thermal loss was 0.4 °C lower for rats treated with the four-inflow setup. Rat core temperatures were kept stable using occasional tail cooling, and rarely exceeded 39 °C. Conclusion: Increasing the number of inflow catheters from one to four resulted in increased flow and temperature homogeneity and stability. Tail cooling is an adequate technique to prevent rats from overheating during 90-min treatments. This validated design can improve accuracy in future in vivo experiments investigating the impact of relevant parameters on the efficacy of different HIPEC protocols.

## 1. Introduction

Peritoneal surface malignancy is an advanced stage disease, generally associated with rapid disease progression and poor prognosis. Most peritoneal surface malignancies are peritoneal metastases, commonly originating from ovarian, gastric or colorectal cancer [1,2]. Treatment options for these patients are limited [3]. Systemic chemotherapy seems to be less effective when malignancies spread to the peritoneum when compared to distant metastases as a result of hematogeneous dissemination, likely related to differences in blood supply and drug penetration. Removing all macroscopic visible tumor has reported effectiveness in the treatment of peritoneal metastases from several primary tumors. Such cytoreductive surgery (CRS) will inherently leave microscopic disease behind, potentially resulting in recurrences. To treat residual tumors, CRS has been combined with hyperthermic intraperitoneal chemotherapy (HIPEC). Immediately after surgery, a heated chemotherapeutic solution is circulated through the abdomen. The elevated temperatures (40–43 °C) enhance the cytotoxic effect of chemotherapeutic drugs, thereby eradicating microscopic disease. Eight different treatment parameters have an impact on the efficacy of HIPEC: type of drug, drug concentrations, carrier solution, volume of the perfusate, temperature of the perfusate, duration of the treatment, delivery technique and patient selection [4]. Up to now, there are no standardized protocols and the biological rationale for specific chemotherapeutic drugs, specific heating periods and specific temperatures is currently incomplete.

Preclinical research can provide insight into the influence of the eight parameters. Several in vitro studies investigated the temperature dependent effectiveness of chemotherapeutic drugs on cancer cells, showing that elevated temperatures contribute to the efficacy of HIPEC [5,6,7,8,9,10,11]. Unfortunately, these in vitro studies are difficult to translate to the clinic since the tumor microenvironment is not accounted for. Furthermore, in vitro experiments do not provide insight into possible toxicity to healthy organs. In vivo studies, in mice, rats and pigs, provide more realistic conditions in which all eight HIPEC parameters can be tested [12,13,14,15,16,17,18,19]. However, establishing the effect of a specific parameter requires that other parameters, like temperature, are well controlled. One of the most elaborate thermal measurements during HIPEC was performed in advanced stage ovarian cancer patients [20], where temperatures were measured at five locations in the abdomen. Temperatures varied between sites and over time with thermal fluctuations up to 4 °C, too high to determine the cytotoxic enhancement locally. Therefore, a pre-clinical set-up that allows a well-controlled thermal distribution is essential to investigate the effect of individual parameters and to optimize HIPEC treatments. Microscopic peritoneal metastases are difficult to locate and, therefore, the entire peritoneal surface should be treated equally to ensure the total eradication of the disease. Therefore, an optimized HIPEC setup should be able to produce uniform drug and temperature distributions for each animal across the entire peritoneal surface.

Numerical modelling can help to optimize the chemotherapy and temperature distributions [21,22]. Recently, we developed treatment planning tools for open HIPEC treatments in rat models, based on computational fluid dynamics (CFD) software [23,24]. The software is able to predict and map flow patterns in the peritoneal cavity for different treatment setups and provide estimations on the systemic drug concentration and core temperature. Results showed that homogeneity can be significantly improved compared to the standard setup of a single inflow and outflow catheter. Maximizing the distance between in- and outflow, the addition of multiple catheters and increased flow rates resulted in increased thermal homogeneity. The optimal catheter setup was found to be four catheters, high flow rate and superficial outflow. Both in vitro [11] and in silico [23,24] studies emphasize the importance of the development of an optimized small animal model.

This study presents the design and validation of a preclinical four-inflow HIPEC setup producing a stable thermal homogeneous flow pattern. The experimental setup was designed using state-of-the-art techniques, including our in-house developed treatment planning tools and 3D printed experimental tools developed using computer-aided design (CAD). The treatment planning tools were used as a predictive tool for flow rate exploration and temperature estimation. Experimental HIPEC treatments were performed on athymic nude rats to compare thermal distributions for a standard one-inflow and a more advanced four-inflow setup. Temperatures were monitored using extensive thermometry and measurements were compared with treatment planning simulations.

## 2. Material and Methods

**Figure 1:**
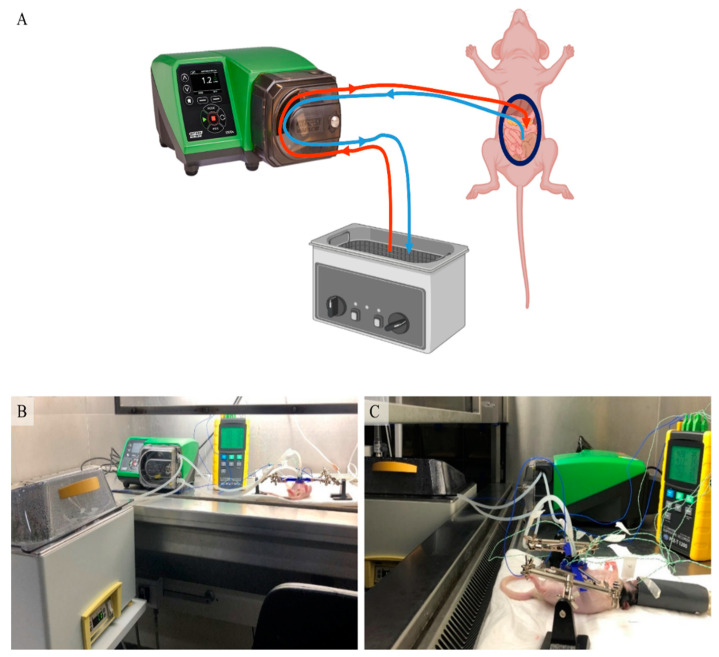
A schematic representation of the hyperthermic intraperitoneal chemotherapy (HIPEC) setup in small animals (**A**). The abdominal wall of a rat is retracted by sutures attached to a plastic retractor. The heated perfusate is stored in a temperature controlled water bath and is circulated by a roller pump. This results in a closed-circuit fluid circulation (**B**,**C**) in which the temperature is measured at eight different locations with a multi-channel data-logging digital thermometer.

**Figure 2:**
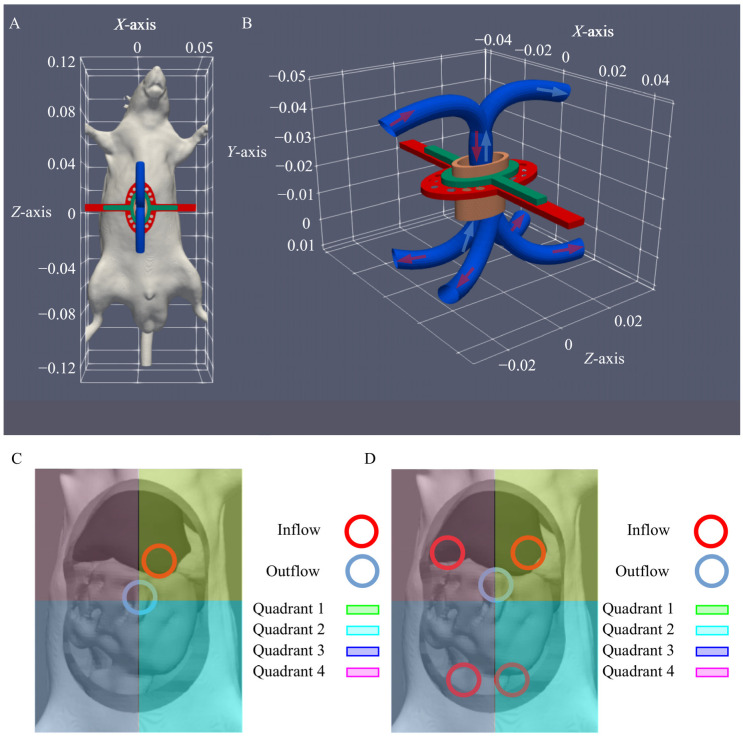
A graphical representation of the semi-open container construction (**A**,**B**). The inflow apparatus features a PVC retractor. Within the retractor, we placed the semi-open container construction, perforated with two holes. The inflow catheter is placed inside the peritoneal cavity through one of the holes. The other hole allows the perfusate to flow freely into the semi-open container construction in which the outflow is placed, ensuring that unobstructed suction is possible. Panel (**C**,**D**) show the catheter placement for the setup using one- and four-inflow(s) catheters, respectively.

In this section we describe the development of the HIPEC setup used for the perfusion in 12 athymic nude rats. A detailed preclinical HIPEC setup (see Figure 1) and protocol for rats is described below. We aimed to evaluate the homogeneity in the peritoneal cavity, subdivided into four quadrants (see Figure 2C) and to ensure that the core temperature of the animals did not overheat. This was evaluated during in silico and in vivo experiments.

### 2.1. Animal Model

We examined thermal distributions in non-cancerous peritoneal cavities of rats. Twelve 6-weeks old female athymic nude rats (Crl: NIH-Foxn1^rnu^) were obtained from Charles River Laboratories Research models and services, Sulzfeld, Germany. Rats were housed in individually ventilated cages (IVC-cages) (two per cage) with corn cob bedding material under standardized conditions: temperature 21 °C, relative humidity 50–60%, 12 h light/12 h dark and with free access to water and food. All animals were acclimatized for 2 weeks before the start of the experiment. HIPEC was performed after 10 weeks with either a standard one-inflow (*n* = 6) or a more advanced four-inflow (*n* = 6) construction. The median weight of the rats on the day of the treatment was 209.5 g. All experiments were approved by the Dutch Central Committee of Animal Experiments with approval code AVD1180020174184, received on 14 February 2019 and carried out in accordance with the Dutch Animal Welfare Act 1997.

### 2.2. HIPEC Setup

A closed-circuit was created by placing two pumpsil tubes through a roller pump (530 Un/R, Watson-Marlow, Barendrecht, The Netherlands) with one end connected to our in-house developed inflow apparatus. The other end was placed in the perfusate stock solution placed in a thermostatically water bath (aqualine AL12, Lauda, Lauda-Königshofen, Germany) (Figure 1A–C). Details of the inflow apparatus are described below. The heated solution was circulated through the abdominal cavity. The temperature was monitored at eight different locations: in the perfusate stock solution, the in- and outflow catheters, in each of the four quadrants and underneath the tongue by using a multi-channel data-logging digital thermometer (PCE-T 1200, PCE-instruments, Enschede, The Netherlands).

### 2.3. Inflow Apparatus

An inflow apparatus was designed using one- or four-inflow catheters and one superficial outflow catheter. One of the challenges during HIPEC in small animal models is ensuring an unobstructed flow for the entire treatment duration. Visceral tissues and fat can easily be sucked up and clog the outflow catheter, especially at higher flow rates. For this reason, we designed a semi-open container construction which effectively separates the outflow region from the peritoneal cavity. The choice of a semi-open setup also improves the thermal homogeneity compared to a completely open setup. The open technique will result in more heat loss compared to the closed technique, therefore a semi-open delivery technique was designed in order to achieve a more constant and stable thermal distribution. The semi-open container construction was designed with 3D software (OpenFOAM; Salome-Meca [25]) and printed using a 3D printer (Builder 3D printer). Figure 2A,B represent the graphical design of the inflow apparatus. The inflow apparatus features a retractor, made out of polyvinyl chloride (PVC) produced by laser cutting technology. To ensure that the semi-open container construction is properly positioned, a click-on system was designed on top of the retractor. The inflow catheter is placed through the opening at the lower compartment of the semi-open container construction to ensure that the inflow is positioned inside the peritoneal cavity below the semi-open construction. The inflow is used as a single catheter (Figure 2C) or is split into four inflow catheters (Figure 2B,D). The second opening allows the perfusate to flow freely into the semi-open container construction in which the outflow is placed and unobstructed suction is possible.

### 2.4. In Silico Flow Rate Exploration

We used the CFD treatment planning software presented in previous reports [23,24] to determine the maximal tolerated flow rate in rats and to provide temperature estimations. We added an insulating term to reflect the lower heat loss through the abdominal opening which was sealed off by the container. The inflow temperature was set at 43 °C and the temperature distribution was evaluated in all four quadrants. Furthermore, we evaluated the core temperature, ensuring that this value did not exceed 39 °C. Flow rates considered were 0.4 mL/s, 0.8 mL/s, 1.2 mL/s and 1.6 mL/s corresponding to 55 rpm, 110 rpm, 165 rpm and 220 rpm for the roller pump. Two catheter setups were considered: a standard one-inflow and a more advanced four-inflow construction (Figure 2C,D). The four-inflow setup was selected because of the results presented in [24], where a four-inflow setup in combination with a high flow velocity was found to result in the most homogeneous temperature distribution. The inflow rates were equally distributed over the 4 catheters.

### 2.5. HIPEC Procedure

**Figure 3:**
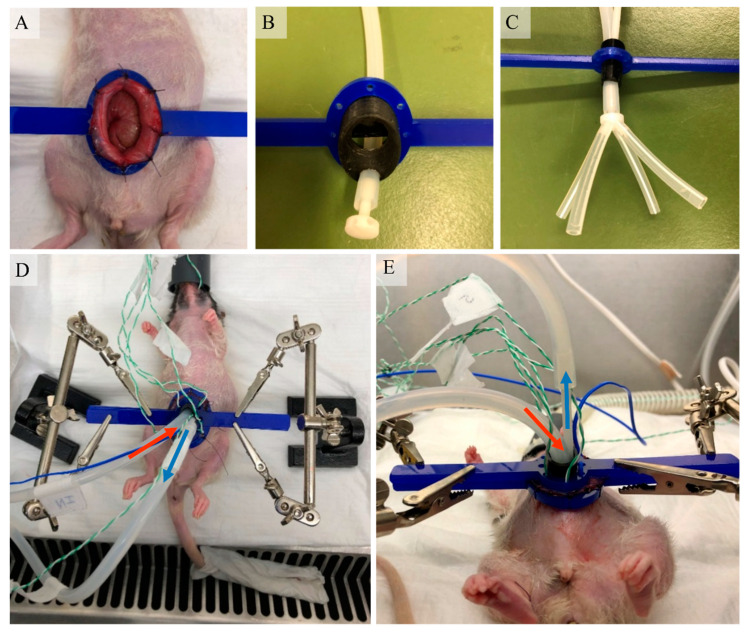
The HIPEC procedure in rats. The abdominal wall was attached to a plastic ring via eight sutures (**A**), enabling the placement of different inflow constructions; the one-inflow (**B**) or the four-inflow (**C**) setup. The plastic ring was placed in two retractor holders to ensure that the construction was properly positioned and to create space in the abdominal cavity and to ensure that the abdomen was filled with the perfusate (**D**,**E**). The temperatures were monitored by thermocouple probes (in blue) placed underneath the tongue, in the in- and outflow catheters and in each of the four quadrants (**D**,**E**).

The HIPEC procedure was performed in a biosafety cabinet. Rats were anesthetized with 0.5–2.5% isoflurane in 100% oxygen using an isoflurane anesthesia vaporizer. To avoid decreasing body temperatures, rats were placed on a heating mat during preparation. To allow access to the peritoneum, the belly was disinfected with betadine and a midline incision of maximum 4 cm was made at the lower part of the abdomen. The abdominal wall was attached to a plastic retractor ring via eight sutures (Figure 3A). Either the one-inflow (Figure 3B) or the four-inflow (Figure 3C) was placed through the semi-open container construction. The inflow was properly positioned in the abdomen before the inflow apparatus was placed on top of the plastic retractor ring. To ensure a proper positioning during the procedure, both the inflow apparatus and retractor were fixed with two retractor holders (Third-hands, Toolcraft, Hirschau, Germany) (Figure 3D,E).

The perfusate (500 mL) was pre-heated using a thermostatically water bath which was set at 49.5 °C, in order to achieve an inflow temperature of 42–43 °C. The heating mat was removed at the start of the HIPEC treatment. After approximately 10–15 min the desired temperature was reached in all four quadrants. Subsequently a 90-min HIPEC procedure was performed. The aim of our pre-clinical HIPEC setup is to achieve a stable temperature distribution for 90 min, since the duration varies between 30–90 min in the clinic. If a stable distribution can be maintained for up to 90 min, the impact of relevant treatment parameters can also be evaluated for protocols with a shorter duration. The abdomen was manually massaged every 15 min during the HIPEC to ensure a uniform delivery of perfusate.

During treatment, the temperature was monitored at the perfusate stock solution, the in- and outflow catheters, and in each of the four quadrants. The core temperature was monitored during the HIPEC procedure using a thermocouple probe underneath the tongue. When the core temperature exceeded 38.5 °C, the body of the rat was partially cooled by placing the tail in a paper tissue soaked with 70% ethanol (Figure 2D).

After the 90-min HIPEC procedure, the inflow catheter was removed from the perfusate and the remaining fluid was removed from the abdomen. The semi-open construction and retractor were removed and the peritoneum was checked for lesions, after which the abdominal wall and the skin were closed with single sutures and disinfected with betadine.

### 2.6. Supportive Care

Before the start of the HIPEC procedure, the animals were treated with carprofen rimadyl (50 mg/mL, Patterson Veterinary, Greeley, CO, USA) to prevent inflammation and reduce postoperative pain. Carprofen was supplied via the drinking water at a concentration of 0.0067 mg/mL, two days in advance until two days after surgery. Immediately after opening of the abdomen the rats were also injected subcutaneously with a high dose of carprofen (5 mg/kg) to increase the systemic carprofen levels. Food and drinking water were made accessible by placing food in the cage and providing a long spout.

## 3. Results

In this section we first discuss the results of the in silico flow rate exploration for the determination of the optimal flow rate for the in vivo experiments. Then we discuss the outcome of the in vivo experiments, focusing on the thermal parameters and the core temperature during HIPEC.

### 3.1. In Silico Flow Rate Exploration

**Figure 4:**
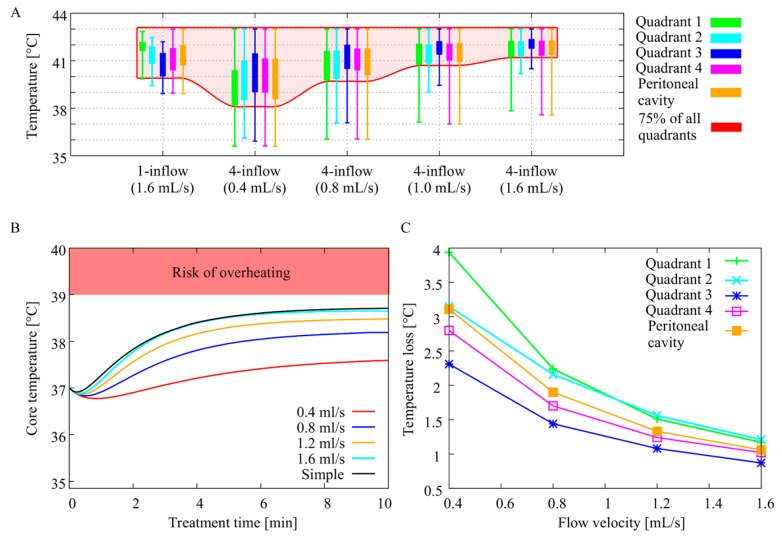
Results of the simulation study of the flow rate exploration. Inflow temperature was set to 43 °C. The interquartile range, minimum and maximum values of the four quadrants are visualized by the boxes and whiskers (**A**). Higher flow rates resulted in higher core temperatures, but these remained below the critical temperature above which overheating becomes a risk (**B**). The flow rate dependency is presented by the thermal loss between inflow and quadrant as a function of the flow rate (**C**).

The thermal distribution predicted by the in silico study is shown in Figure 4A. The boxes show the interquartile range while the whiskers show the minimum and maximum values in the four quadrants. Cold spots near the fluid surface are impossible to avoid, and thus a low(er) minimum value is expected. Therefore, we compared the temperature level at which 75% of the volume, in each quadrant, is heated sufficiently as depicted by the shaded area in Figure 4A. The four-inflow apparatus does not result in a benefit compared to the one catheter setup if 0.4 mL/s is used. Doubling the flow velocity resulted in an increase of 1.7 °C of the 75% region compared to the four-inflow setup with 0.4 mL/s. Further increasing the flow rate resulted in an additional increase of 1.3 °C. Using 1.6 mL/s, 75% of the volume in all quadrants was between 41 °C and 43 °C. The flow rate dependency is emphasized in Figure 4C, showing the thermal loss between the inflow and each quadrant as a function of the flow rate. In Figure 4B, we show the simulated core temperature for all flow rates and inflow setups. Higher flow rates resulted in higher core temperatures, but these remained below the critical temperature (39 °C) above which overheating becomes a risk. Therefore, from the in silico study we can conclude that a flow rate of 1.6 mL/s, using the inflow apparatus from Figure 2B, is a safe and adequate way of delivering a homogeneous HIPEC treatment.

### 3.2. In Vivo Temperature Distribution

We performed 90-min HIPEC treatments with phosphate-buffered saline (PBS) on athymic nude rats. One group of rats was treated with the one-inflow constructions and the second group was treated with the four-inflow construction. Both groups were treated with a flow rate of at 1.6 mL/s (6 rats/group). After the HIPEC procedures, the entire peritoneum was checked and no lesions were observed. During these experiments the temperature was monitored at the stock solution, in- and outflow catheters, in each of the four quadrants and underneath the tongue.

**Figure 5:**
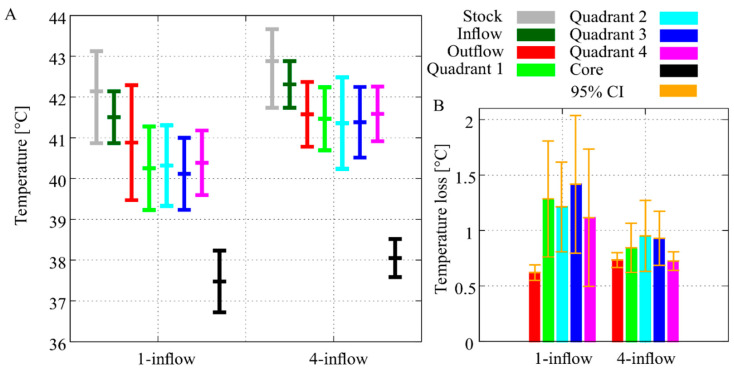
Monitored temperatures during the 90-min HIPEC procedure performed on rats with a Figure 1. 6 mL/s. The HIPEC treatment with a one-inflow construction results in lower temperatures at all measured locations compared to the four-inflow construction (**A**). The temperature loss between inflow and the outflow/quadrants, demonstrating less temperature loss for the four-inflow compared to the one-inflow (**B**). Temperature loss between mean inflow and mean quadrant/outflow temperatures, averaged over treatment duration and over the six rats per setup. The orange whiskers depict the 95% confidence interval between rats.

Figure 5A shows the mean ± SD during the 90-min HIPEC perfusion. The inflow temperature was 41.5 °C ± 0.64 (mean ± SD) and 42.3 °C ± 0.57 (mean ± SD) for the one- and four-inflow construction, respectively. The inflow temperature decreased due to thermal losses and instability in the peritoneal cavity of the rat, consequently impacting the stock solution and inflow temperature as a result of the closed-circuit used during the experiments. Water bath conditions were kept constant for all experiments to compare the effect of both setups such that we did not compensate for the thermal losses observed for the one-inflow setup. In both cases the outflow temperature was lower than the inflow temperatures, 40.9 °C ± 1.41 vs. 41.6 °C ± 0.79 (mean ± SD). Comparing the temperature in the quadrants, the four-inflow clearly shows a beneficial effect. This is emphasized by the temperature loss between inflow and quadrant temperatures (Figure 5B). The thermal loss in each quadrant was around 0.4 °C lower when the four-inflow setup was used.

**Figure 6:**
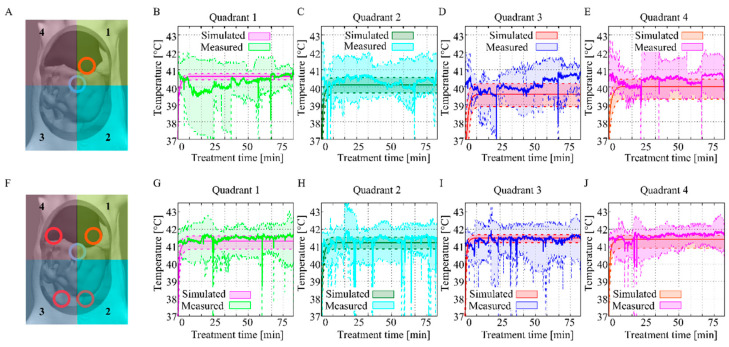
Comparison between the in silico, in vivo data and the one-inflow (**A**) and four-inflow (**F**) setups. The simulations were performed with an inflow temperature equal to the mean inflow temperature during the measurements, as presented in Figure 5A. The mean measured temperatures with the one-inflow compare well with the simulated data (**B**–**E**). The instability of the one-inflow setup is the main reason for deviations. The measured data for the four-inflow setup is within the simulated data range (**G**–**J**). The inflow temperature for these simulations were set to the measured data during experiments, to ensure an equitable comparison. Temperatures are much more stable over time during treatments performed with the four-inflow setup.

In Figure 6 the results of the in silico and the in vivo data for the one-inflow (Figure 6A–E) and the four-inflow (Figure 6 F–J) setups are combined. The simulated data of Q1–Q4 are represented by the colors pink, green, red and orange, respectively. The shaded area shows the interquartile range within the volume of a quadrant. The data of Q1–Q4 measured in six rats per inflow setup are presented in green, light blue, dark blue and pink, respectively. The shaded area shows the minimum and maximum values. Measured data match the simulated data for the four-inflow setup since the mean of the measured data is within the interquartile range of the simulated data. The data of the one-inflow construction differ in the first quadrant but are similar in the other three quadrants. In general, the in silico and in vivo data correspond well and we can conclude that the use of the four-inflow construction is able to realize a more homogeneous and stable flow with less fluctuations.

### 3.3. Core Temperature

**Figure 7:**
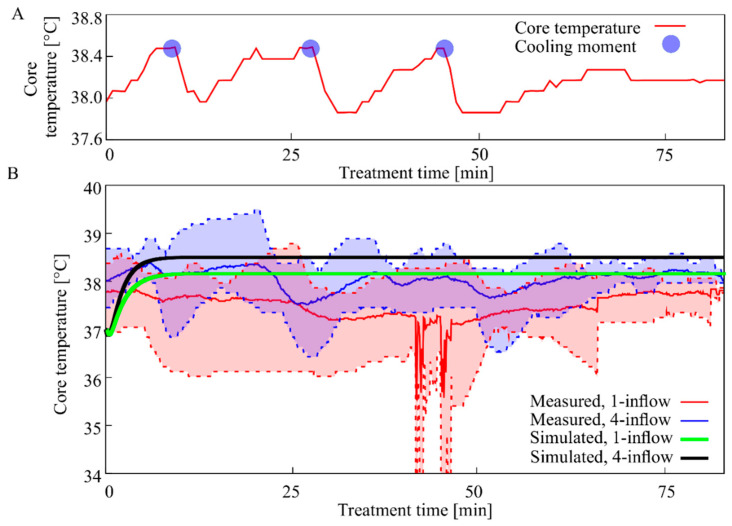
Core temperature measurements and simulations. A typical example of the rat’s core temperature during a 90-min HIPEC treatment with four-inflow setup (**A**). The blue dots indicate the moments an ethanol soaked paper tissue was wrapped around the rat’s tail. The measured and simulated core temperature during the HIPEC procedures with the one-inflow (red and green) and four-inflow (blue and black) construction are presented (**B**). The plot represents the mean of 6 rats with the minimum and maximum measured temperatures (dotted) for the one-inflow setup (red) and four-inflow setup (blue), the simulated core temperature are visualized by the green (one-inflow) and black (four-inflow) lines. The simulations were performed with an inflow temperature equal to the mean inflow temperature during the measurements, as presented in Figure 5A.

The core temperature of untreated rats is 37–38 °C and it should not exceed 39 °C. The core temperature in rats receiving the one-inflow HIPEC setup was lower than in rats treated with the four-inflow setup, 37.5 °C ± 0.76 vs. 38 °C ± 0.47 (mean ± SD) (Figure 5). The highest average core temperature was measured during treatment with the four-inflow construction (39.4 °C), but cooling the rat’s tail prevented overheating. The core temperature was more stable when using the four-inflow setup compared to the one-inflow setup. Figure 7A gives a typical example of the core temperature of a rat during a 90-min HIPEC treatment with the four-inflow. The blue dots represent the moments at which an ethanol soaked paper tissue was wrapped around the rat’s tail. The immediate drop in core temperature shows that this is an adequate cooling method. The mean of the measured core temperatures of all rats over time during the treatment (red and blue) do not exceed the simulated core temperatures (green and black) (Figure 7B).

## 4. Discussion

This study describes a dedicated preclinical in vivo HIPEC setup in rats. Based on results from in silico experimental data, a four-inflow setup was developed to improve the homogeneity of the temperature distribution in the abdomen. In vivo experiments demonstrated that increasing the number of inflow catheters from one to four resulted in a homogeneous and stable temperature distribution which is key for an optimal HIPEC procedure suitable for in vivo HIPEC research under well-controlled conditions.

The temperature settings for the water bath were kept constant to compare the effect of both inflow constructions on the thermal flow. Due to fact that we used a closed circuit, larger thermal losses and thermal instability caused a drop in inflow temperature for the one-inflow setup. In Figure 5B, the temperature loss between the inflow and quadrant temperature is demonstrated. In general, using the four-inflow setup decreased the temperature loss by approximately 0.4 °C, corresponding to a significant increase in homogeneity. The standard deviation was lower for seven out of eight measurement points for the four-inflow setup, which is expected since higher temperatures lead to higher deviations. Our data confirm that using the four-inflow catheter setup results in a more stable and higher mean temperature in the abdominal quadrants (Figure 6). Reduced stability is also caused by thermal loss in the stock solution. The significant heat loss in the rats treated with the one-inflow setup resulted in a drop in temperature in the stock solution and in the inflow temperature. Stability was easily maintained during the treatments using the four-inflow setup.

The core temperature of the rats was well controlled during the treatment. Cooling of the rat’s tail resulted in a direct and steep drop in core temperature when needed. The maximum core temperature was generally below 39 °C, as shown in Figure 7B. Therefore, we conclude that the setup designed for this study is able to produce a homogeneous, safe and stable thermal distribution in a small animal model.

The roller pump used in this study had a maximum rotation rate of 220 rpm, yielding a flow of 1.6 mL/s, which was therefore the maximum flow rate considered in this study. We expect that when higher flow rates are used, even more homogeneity and higher median temperatures can be reached. The flow rate is then only limited by the maximally tolerable flow, increase in core temperature and the experimental feasibility since it becomes more difficult to produce an unobstructed continuous flow for high(er) flow rates.

A direct comparison between the experimental data and the simulated data is presented in Figure 6. Simulated and measured data compare well, especially for the four-inflow setup. Comparing the simulated data and the experimental data for the one-inflow case, there are slight discrepancies. This can be explained by the influence of manual massaging, which is difficult to simulate, and therefore not accounted for. The effect of massaging is largest when the heated fluid is injected into one quadrant (Q1). Compared to the measured data, the temperature in quadrant one is slightly higher and in the other quadrants slightly lower. The massaging redistributes this heat, visible in the measured data. This effect is not significant for the four-inflow setup, where the heat is distributed more uniformly. Treatment using a four-inflow setup is therefore more stable, and thus better predictable by simulation. Nevertheless, all simulated data compare well to the measured data.

The homogeneous and stable thermal distribution, as realized with this four-inflow setup, is important in future in vivo HIPEC research. Eight cross dependable parameters can affect the efficacy of a HIPEC treatment. One of landmark clinical papers, the randomized controlled trial on CRS/HIPEC in colorectal cancer by Verwaal et al. [26], already mentioned that questions about several aspects regarding the delivery of HIPEC, such as the best choice of drug and dose, remain completely open. The PRODIGE-7 trial [27] in colorectal cancer patients suggests that composing the appropriate HIPEC regime is crucial. All patients received systemic chemotherapy before randomization, mainly oxaliplatin based. The results showed no survival benefit for patients treated with CRS and HIPEC compared to patients treated with CRS only. A 30-min oxaliplatin-based HIPEC at 42–43 °C was used. This study sparked a debate, but ineffectiveness of an oxaliplatin-based HIPEC in this setting might be explained by desensitizing induction chemotherapy, a high dose of glucose in the carrier solution and short treatment time [28,29]. This underlines the importance of firm scientific evidence for the choice of parameters and a setup as presented in this paper provides an excellent basis for preclinical research to answer such clinical questions. Furthermore, techniques described in this study, such as the treatment planning tool, computer aided design, variation of catheter number and positions, flow rate variation, can be translated to the clinic to optimize the treatment setups used in humans as well. This is part of ongoing research in our group.

The temperature is one of the important parameters to be optimized. In 2001, a Japanese group [30] performed a three-armed randomized controlled trial comparing normothermic chemoperfusion with surgery, hyperthermic chemoperfusion with surgery and surgery alone, performed on 139 patients diagnosed with T2–4 gastric cancer. They found that the five year survival rate was similar for patients treated with normothermic chemoperfusion or surgery alone (43% vs. 42%). However, the five year survival rate was higher for patients treated with hyperthermic chemoperfusion (61%). Therefore, it is justified to assume that the locally achieved temperature does have an impact on the clinical outcome. A recent in vitro study performed by our group [11] also indicated that the efficacy of the chemotherapy strongly depends on the locally reached temperatures. The temperature distribution determines the efficiency of the other key parameters, which determines the cytotoxic enhancement of chemotherapies, the required dose and the maximum treatment duration.

The optimized preclinical animal model presented in this study is a first step towards thermal homogeneity and offers a stable model to examine other treatment parameters and determine the optimal choice. For example, comparing chemotherapies on a certain type of tumor only makes sense when the enhancement is equal across the entire peritoneal surface. Alternatively, various treatment durations can only be compared when a stable flow for the entire duration of a treatment can be guaranteed. By using dedicated in vivo models, results from preclinical research focused on determining the optimal parameter choices can become pivotal in future clinical trials.

## 5. Conclusions

Our data provide a validated, comprehensive design and protocol of a preclinical in vivo HIPEC setup in rats. The HIPEC setup used in this study is able to generate uniform and well controlled temperature distributions in the peritoneal cavity of a rat, necessary to study the effect of relevant HIPEC parameters. Increasing the number of inflow catheters from one to four resulted in increased homogeneity and stability. Rat tail cooling is an adequate technique to prevent systemic overheating during 90-min treatment. The techniques described in this study can help in future in vivo experiments investigating the impact of various parameters on the efficacy of HIPEC treatments.

## References

[1] Flanagan M., Solon J., Chang K., Deady S., Moran B., Cahill R., Shields C., Mulsow J. (2018). Peritoneal metastases from extra-abdominal cancer—A population-based study. Eur. J. Surg. Oncol..

[2] Desai J.P., Moustarah F. (2020). Cancer, Peritoneal Metastasis.

[3] Sugarbaker P.H. (1988). Surgical management of peritoneal carcinosis: Diagnosis, prevention and treatment. Langenbeck’s Arch. Surg..

[4] Helderman R.F., Löke D.R., Kok H.P., Oei A.L., Tanis P.J., Franken N.A.P., Crezee H. (2019). Variation in Clinical Application of Hyperthermic Intraperitoneal Chemotherapy: A Review. Cancers.

[5] Murata S., Yamamoto H., Shimizu T., Naitoh H., Yamaguchi T., Kaida S., Takebayashi K., Miyake T., Tani T., Tani M. (2018). 5-fluorouracil combined with cisplatin and mitomycin C as an optimized regimen for hyperthermic intraperitoneal chemotherapy in gastric cancer. J. Surg. Oncol..

[6] Urano M., Ling C.C. (2002). Thermal enhancement of melphalan and oxaliplatin cytotoxicity in vitro. Int. J. Hyperth..

[7] Kjellström J., Kjellén E., Johnsson A. (2005). In vitro radiosensitization by oxaliplatin and 5-fluorouracil in a human colon cancer cell line. Acta Oncol..

[8] Ubink I., Bolhaqueiro A.C.F., Elias S.G., Raats D.A.E., Constantinides A., Peters N.A., Wassenaar E.C.E., De Hingh I.H.J.T., Rovers K.P., Van Grevenstein W.M.U. (2019). Organoids from colorectal peritoneal metastases as a platform for improving hyperthermic intraperitoneal chemotherapy. BJS.

[9] Jung H. (1982). Interaction of thermotolerance and thermosensitization induced in CHO cells by combined hyperthermic treatments at 40 and 43 degrees C. Radiat. Res..

[10] Van Der Heijden A.G., Jansen C.F., Verhaegh G., O’Donnell M.A., Schalken J.A., Witjes J.A. (2004). The Effect of Hyperthermia on Mitomycin-C Induced Cytotoxicity in Four Human Bladder Cancer Cell Lines. Eur. Urol..

[11] Helderman R.F., Löke D.R., Verhoeff J., Rodermond H.M., Van Bochove G.G., Boon M., Van Kesteren S., Vallejo J.J.G., Kok H.P., Tanis P.J. (2020). The Temperature-Dependent Effectiveness of Platinum-Based Drugs Mitomycin-C and 5-FU during Hyperthermic Intraperitoneal Chemotherapy (HIPEC) in Colorectal Cancer Cell Lines. Cells.

[12] Lemoine L., Thijssen E., Carleer R., Cops J., Lemmens V., Van Eyken P., Sugarbaker P., Van Der Speeten K. (2019). Body surface area-based versus concentration-based intraperitoneal perioperative chemotherapy in a rat model of colorectal peritoneal surface malignancy: Pharmacologic guidance towards standardization. Oncotarget.

[13] Pelz J., Doerfer J., Hohenberger W., Meyer T. (2005). A new survival model for hyperthermic intraperitoneal chemotherapy (HIPEC) in tumor-bearing rats in the treatment of peritoneal carcinomatosis. BMC Cancer.

[14] Lehmann K., Rickenbacher A., Jang J.-H., Oberkofler C.E., Vonlanthen R., Von Boehmer L., Humar B., Graf R., Gertsch P., Clavien P.-A. (2012). New Insight into Hyperthermic Intraperitoneal Chemotherapy. Ann. Surg..

[15] Klaver Y.L.B., Hendriks T., Lomme R.M.L.M., Rutten H.J.T., Bleichrodt R.P., De Hingh I.H.J.T. (2011). Intraoperative versus Early Postoperative Intraperitoneal Chemotherapy after Cytoreduction for Colorectal Peritoneal Carcinomatosis: An Experimental Study. Ann. Surg. Oncol..

[16] Shimizu T., Maeta M., Koga S. (1991). Influence of local hyperthermia on the healing of small intestinal anastomoses in the rat. BJS.

[17] Sánchez-García S., Padilla-Valverde D., Villarejo-Campos P., García-Santos E.P., Martín-Fernández J. (2017). Hyperthermic chemotherapy intra-abdominal laparoscopic approach: Development of a laparoscopic model using CO2 recirculation system and clinical translation in peritoneal carcinomatosis. Int. J. Hyperth..

[18] Sánchez-García S., Padilla D., Villarejo-Campos P., Martín-Fernández J., García-Rojo M., Rodríguez-Martínez M. (2014). Experimental development of an intra-abdominal chemohyperthermia model using a closed abdomen technique and a PRS-1.0 Combat CO2 recirculation system. Surgery.

[19] Gesson-Paute A., Ferron G., Thomas F., De Lara E.C., Chatelut E., Querleu D. (2007). Pharmacokinetics of Oxaliplatin During Open Versus Laparoscopically Assisted Heated Intraoperative Intraperitoneal Chemotherapy (HIPEC): An Experimental Study. Ann. Surg. Oncol..

[20] Rettenmaier M.A., Mendivil A.A., Gray C.M., Chapman A.P., Stone M.K., Tinnerman E.J., Goldstein B.H. (2015). Intra-abdominal temperature distribution during consolidation hyperthermic intraperitoneal chemotherapy with carboplatin in the treatment of advanced stage ovarian carcinoma. Int. J. Hyperth..

[21] Kok H.P., Beck M., Löke D.R., Helderman R.F.C.P.A., Van Tienhoven G., Ghadjar P., Wust P., Crezee H. (2020). Locoregional peritoneal hyperthermia to enhance the effectiveness of chemotherapy in patients with peritoneal carcinomatosis: A simulation study comparing different locoregional heating systems. Int. J. Hyperth..

[22] Schooneveldt G., Löke D.R., Zweije R., Helderman R.F., Kok H.P., Crezee H. (2020). Experimental validation of a thermophysical fluid model for use in a hyperthermia treatment planning system. Int. J. Heat Mass Transf..

[23] Löke D.R., Helderman R.F.C.P.A., Franken N.A.P., Oei A.L., Crezee J., Kok H.P. (2020). Simulating drug penetration during hyperthermic intraperitoneal chemotherapy. Drug Deliv..

[24] Löke D.R., Helderman R.F.C.P.A., Rodermond H.M., Tanis P.J., Streekstra G.J., Franken N.A.P., Oei A.L., Crezee J., Kok H.P. (2020). Demonstration of treatment planning software for hyperthermic intraperitoneal chemotherapy in a rat model. Int. J. Hyperth..

[25] Ribes A., Caremoli C. Salomé platform component model for numberical simulation. Proceedings of the 31st Annual International Computer Software and Applications Confernce (COMPSAC 2007).

[26] Verwaal V.J., Bruin S., Boot H., Van Slooten G., Van Tinteren H. (2008). 8-Year Follow-up of Randomized Trial: Cytoreduction and Hyperthermic Intraperitoneal Chemotherapy Versus Systemic Chemotherapy in Patients with Peritoneal Carcinomatosis of Colorectal Cancer. Ann. Surg. Oncol..

[27] Quenet F., Elias D., Roca L., Goere D., Ghouti L., Pocard M., Facy O., Arvieux C., Lorimier G., Pezet D. (2018). A UNICANCER phase III trial of hyperthermic intra-peritoneal chemotherapy (HIPEC) for colorectal peritoneal carcinomatosis (PC): PRODIGE 7. J. Clin. Oncol..

[28] Ceelen W.P. (2019). HIPEC with oxaliplatin for colorectal peritoneal metastasis: The end of the road?. Eur. J. Surg. Oncol..

[29] Nagourney R.A., Evans S., Tran P.H., Nagourney A.J., Sugarbaker P.H. (2020). Colorectal cancer cells from patients treated with FOLFOX or CAPOX are resistant to oxaliplatin. Eur. J. Surg. Oncol..

[30] Yonemura Y., De Aretxabala X., Fujimura T., Fushida S., Katayama K., Bandou E., Sugiyama K., Kawamura T., Kinoshita K., Endou Y. (2002). Intraoperative chemohyperthermic peritoneal perfusion as an adjuvant to gastric cancer: Final results of a randomized controlled study. Hepatogastroenterology.

